# Application of an Endodontic Static Guide in Fiber Post Removal from a Compromised Tooth

**DOI:** 10.1155/2023/7982368

**Published:** 2023-09-15

**Authors:** Mehran Farajollahi, Omid Dianat, Samaneh Gholami, Shima Saber Tahan

**Affiliations:** ^1^Iranian Center for Endodontic Research, Research Institute of Dental Sciences, School of Dentistry, Shahid Beheshti University of Medical Sciences, Tehran, Iran; ^2^Division of Endodontics, Department of Advanced Oral Sciences and Therapeutics, School of Dentistry, University of Maryland, Baltimore, MD, USA; ^3^Department of Restorative Dentistry, School of Dentistry, Shahid Beheshti University of Medical Sciences, Tehran, Iran; ^4^Department of Endodontics, School of Dentistry, Shahid Beheshti University of Medical Sciences, Tehran, Iran

## Abstract

Removing a fiber post from a root canal that requires endodontic retreatment is often very challenging. Conventional freehand techniques for removing fiber posts are time-consuming, sometimes result in iatrogenic errors, and heavily rely on the practitioner's experience. The endodontic static guide can be an alternative method. While the use of an endodontic 3D-printed static guide for fiber post removal has been reported as highly successful, it can also cause complications. Skipping any critical steps during the guide construction or its clinical application can lead to errors. This case report presents the saving of a compromised tooth with a fractured fiber post and a periapical lesion around the apex through the use of an endodontic static guide for fiber post removal. This study describes possible sources of error that may happen during construction and clinical use of the guide.

## 1. Introduction

Composite build-ups reinforced with fiber posts are being used to restore teeth with short clinical crowns in the aesthetic zone [1]. The process of bonding fiber posts inside the root canal involves the use of adhesives like composite resins or glass ionomers, which are known for being challenging to remove [2]. Root canal retreatment in such complex endodontic cases can lead to deviation from the original anatomy of the root canal, perforation, and even irreparable errors [3]. Currently, guided endodontics has become popular for the treatment of calcified root canals [4], dental anomalies such as dens invagination [5, 6], the removal of root canal barriers like glass fiber posts [7] and mineral trioxide aggregate [8], and microsurgical endodontic procedures [9, 10]. The guided-endodontic technique combines the three-dimensional data derived from cone-beam computed tomography (CBCT) devices with the surface data of teeth gathered using an intraoral scanner to create a guide for treatment through 3D printing [11, 12]. The guide will be adjusted to fit the surface of the tooth, thus enabling the drill to access the root canal [13]. In this novel method of treatment, the technology is combined with endodontics for greater precision to avoid unnecessary removal of healthy tooth structure and subsequent complications. This leads to an improvement in the prognosis of the treatment and allows for predictable outcomes with a shorter treatment time, resulting in higher patient satisfaction [14, 15].

Hence, this article presented a case report on utilizing the static-guided endodontic technique to remove a fiber post from a compromised, infected maxillary central incisor of a young patient.

## 2. Case Report

**Figure 1:**
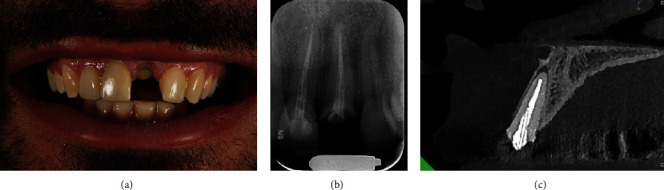
(a) Perioperative clinical examination showing a fractured tooth #9. (b) Perioperative periapical radiograph showing inadequate endodontic treatment with a fractured fiber post in the root canal. (c) Perioperative CBCT assessment. The sagittal slice of tooth #9 confirms a radiolucent area with an intact buccal and palatal plate.

A 28-year-old male patient, with no medical history, presented to the postgraduate endodontic clinic at Shahid Beheshti University of Medical Sciences expressing dissatisfaction with the aesthetic appearance of a fractured maxillary left central incisor. The patient did not express a history of pain. Tooth #9 showed normal mobility upon clinical examination, and its periodontal probing depths were found within normal limits (Figure 1(a)). Furthermore, the tooth was not tender to percussion. A periapical radiograph revealed a fractured fiber post and poor endodontic treatment, causing a periapical lesion around the apex of the root (Figure 1(b)).

A CBCT scan was performed using the NewTom VGi (NewTom, Verona, Italy) operating at 110 kVp, 11.21 mA, with a FOV of 6^∗^6 cm and a voxel size 0.15 mm to obtain a comprehensive view of tooth #9 and the surrounding structures. The imaging showed a radiolucent area with intact buccal and palatal plate (Figure 1(c)). A diagnosis of previous endodontic treatment with asymptomatic apical periodontitis was reached. Due to the high risk of iatrogenic damage to the root while removing the fiber post, the application of a 3D-printed guide was presented to the patient, and informed consent was obtained.

**Figure 2:**
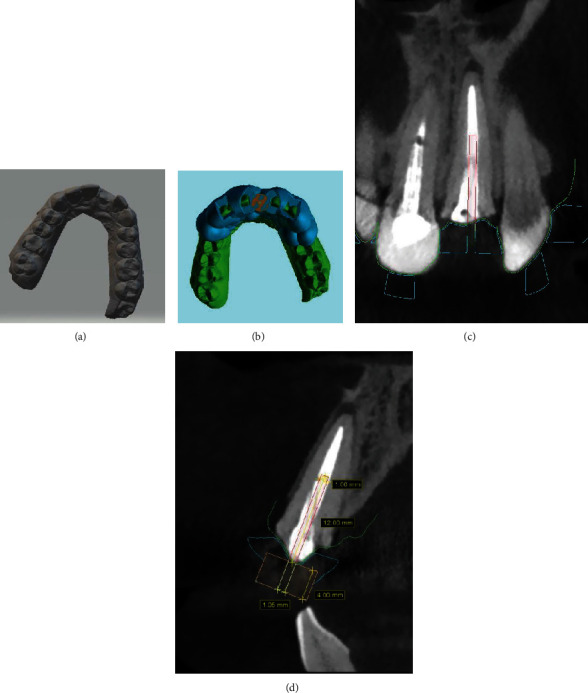
(a) Optical impression of the maxillary arch using STL format. (b) The STL file and the DICOM file obtained by CBCT are virtually merged. (c) Virtual planning of fiber post removal in the coronal plane and (d) sagittal plane. The drill was positioned along the long axis of the fiber post set up to its apical tip.

**Figure 3:**
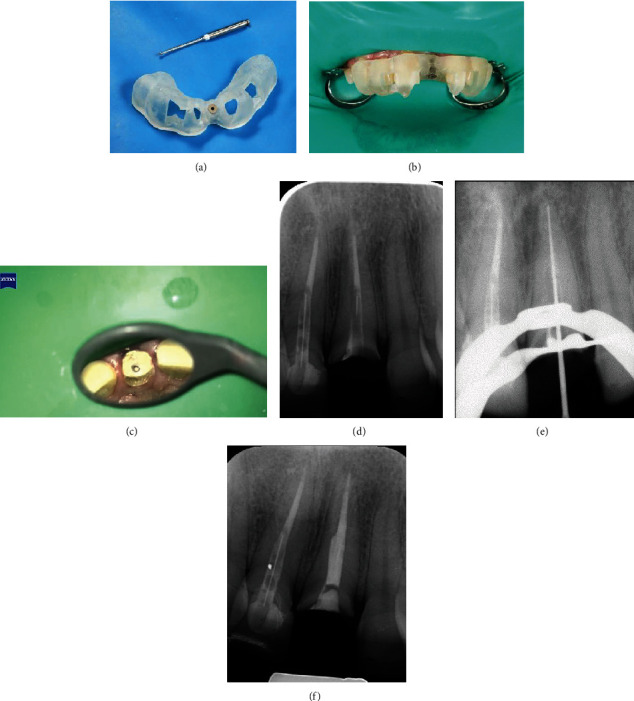
(a) Munce bur and static endodontic guide with metallic sleeve. (b) The guide was positioned on the teeth to check the correct fitting. (c) A microscopic view showing a check on the drilling axis towards the gutta-percha. (d) Periapical radiograph indicates some deviation from the intended pathway. (e) Working length determination radiograph. (f) Final radiograph with temporary filling.

An intraoral scan was taken from the upper arch using the CS 3600 scanner (Carestream Health, Atlanta, Georgia, USA) (Figure 2(a)). Then, both the Standard Tessellation Language (STL) file obtained by optical impression and the DICOM file obtained by CBCT were merged using Blue Sky Plan software (Blue Sky Bio®). The software automatically superimposed the two mentioned files (Figure 2(b)). A virtual path of the bur was designed according to the location, diameter, and apical extent of the fiber post to reach the gutta-percha. A measurement of 12 mm was determined. The virtual path was positioned three-dimensionally along the axis of the fiber post (Figures 2(c) and 2(d)). A guidance hole was created in the location where the metal sleeve would be placed to assist in guiding the drill, and its dimensions were measured. The template was extended to cross the midline for maximal stability. Moreover, verification windows were included in the virtual guide to verify the precise fitting along the dental surfaces during the clinical period. The virtually designed guide was printed by a Digident Quick 3D printer (Mobtakeran Mechatronics Ark Company, Tehran, Iran) with a thickness of 3 mm and an offset of 2 mm using a photopolymerized biocompatible polymer resin (PowerResins SG, Singapore). The customized metal sleeve with a 3 mm external diameter, 1.05 mm internal diameter, and 4 mm length was inserted and held by friction into the previously designed area. Munce bur (Meisinger, Germany) size #1 was used which had a length of 16 mm and a diameter of 1 mm in performing this process (Figure 3(a)).

On the second visit, the guide was precisely positioned in the area, and proper placement was confirmed by examining through the windows. After administering local anesthesia, a rubber dam was put in place with the split dam technique to isolate nine teeth. The correct fitting of the guide was verified again (Figure 3(b)). The drill was inserted with an up-and-down movement (40,000 rpm) from the metal sleeve under irrigation. The entire process of drilling was completed in three stages until exposing the gutta-percha (Figure 3(c)). The complete process was carried out by an endodontic resident. During each step of the process, the guide was taken off, and normal saline was used to irrigate the root canal. Additionally, the drill was also cleaned. After cleaning the debris from the canal using this method, the drilling path was also examined using an operative microscope (OPMI Pico™ Zeiss, Oberkochen, Germany). After the completion of drilling, the periapical radiograph showed some deviation from the intended path (Figure 3(d)). According to the previous design, the total length of the drill was used in the guide, of which 4 mm was engaged in the metal sleeve and 12 mm was in the root canal. This deviation from the root canal did not pose a problem for continuing treatment, and the apical end of the canal was accessible.

**Figure 4:**
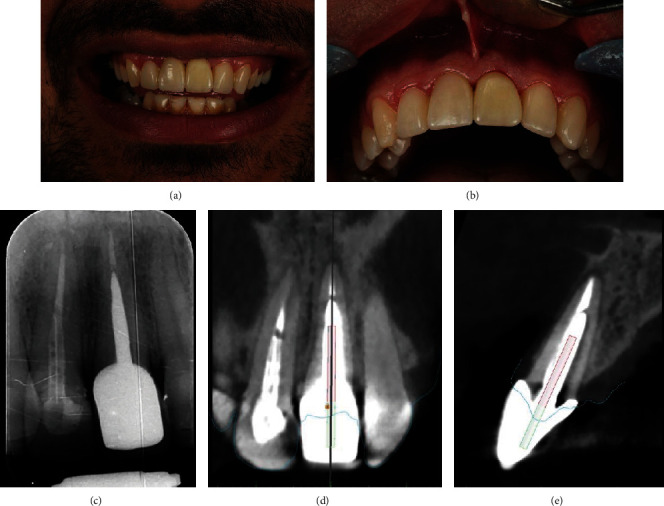
(a) Postoperative clinical view of a permanent restoration. (b) Clinical view of 6 months postoperatively. (c) Radiograph view of 6 months postoperatively showing periapical healing. (d, e) Coronal and sagittal view of postoperative CBCT showing the dimensional reduction of the periapical lesion. The superimposed preplanned pathway is located in the center of the canal.

A size 25 K-file was used along with chloroform to remove the remaining gutta-percha. A size 20 K-file was placed in the canal for length determination using an electronic apex locator (Root ZX; J Morita, Tokyo, Japan), and this length was confirmed by a radiograph (Figure 3(e)). After work length determination utilizing an electronic apex locator (Root ZX; J Morita, Tokyo, Japan), final shaping and cleaning were performed using the S1 and S2 ProTaper Gold system (Dentsply, Ballaigues, Switzerland) along with a 5.25% NaOCl irrigation. The final irrigation protocol was performed by sonic activation (EndoActivator™, Dentsply, Ballaigues, Switzerland), starting with 5.25% NaOCl and then followed by EDTA at 17% and normal saline. The canal was obturated using the warm vertical condensation technique along with an AH-26 (Dentsply Sirona, Ballaigues, Switzerland) root canal sealer. Finally, the tooth was temporarily restored with light-cure glass ionomer cement (GC Fuji II LC, Tokyo, Japan) and referred for a permanent restoration (Figure 3(f)). Due to the significant amount of tooth loss, it was decided to use a casting post and core. Finally, due to the aesthetic zone, the tooth was restored with a full ceramic zirconia crown (Figure 4(a)). At the 6-month recall appointment, the patient's healing progress was assessed through a clinical examination and follow-up periapical radiograph. The tooth was asymptomatic (Figure 4(b)), and the radiograph revealed successful healing of the apical area (Figure 4(c)). CBCT was carried out according to the parameters of previous exposure. CBCT revealed a decrease in the dimensions of the periapical lesion. Due to the placement of the metallic post inside the root canal and the CBCT artifact, it was not possible to calculate the deviation from the intended path (Figures 4(d) and 4(e)).

## 3. Discussion

Fiber post removal from a tooth that requires endodontic retreatment can be challenging. The operator's experience is a crucial factor in the success of conventional free-handed techniques, and the use of an operating microscope may be necessary [16]. Despite the introduction of dental operating microscopes and different techniques to remove fiber posts such as drilling with long-shank burs, ultrasonic tips, or special kits, there are still risks including root perforation, axis deviation, loss of dentin, and subsequent weakening of the roots [17–19]. In this case, due to the need for intracanal retention, apical surgery was not an alternative treatment option; thus, a guided approach was planned to retreat the tooth.

The guided endodontic technique can accurately remove the fiber post, and this approach is predictable. Previous studies reported successful case management of infected root canals with fiber post using endodontic static guides [20, 21]. Perez et al. indicated that the mean deviation between the virtual design and actual dimensions after removing the fiber post was 0.39 ± 0.14 mm coronally and 0.40 ± 0.19 mm apically [7]. Compared to conventional methods, the increased radiation dose and cost associated with using preoperative CBCT and the template can be justified based on the reduced likelihood of iatrogenic errors [14]. Although some articles present promising results for the guided endodontics technique [22, 23], it is possible that clinicians may encounter challenges and errors by using this technique [14]. There is one report of root perforation when performing guided endodontic access in a severely calcified root canal [3]. The use of this technique is not recommended in patients with limited mouth opening [3], but a sleeveless guide system was described that can be used in these patients [24]. A sleeveless endodontic guide is designed to navigate the head of the handpiece instead of the drill. According to Mo et al., the sleeveless guide system demonstrated enhanced accuracy over the conventional freehand method for removing fiber posts [25]. It is crucial to pay attention to every step of designing and printing an endodontic static guide, as well as during its clinical application. Each stage is significant as it may influence the precision of the guide.

The unique settings, sensors, and software used for rendering CBCT devices can either improve or limit a clinician's capacity to appropriately plan this treatment in the presence of artifacts. The NewTom device utilized in this case report appears to exhibit a superior capacity for producing more cleaner images with reduced beam hardening artifacts when compared to other CBCT devices [26]. Furthermore, collimating the primary X-ray beam limits radiation exposure in the desired area and helps to avoid beam hardening artifacts in susceptible regions [27]. Performing a high-resolution scan of the patient's region of interest using a limited field of view and saving it in the DICOM file format is necessary [28].

The accuracy of models in the digital planning process is improved through the use of images obtained by intraoral scanners in the STL file format. New scans must be necessary if the DICOM and STL files are of poor quality [29]. No clinical alterations to the position and morphology of the teeth should be made after the CBCT and intraoral scanning processes [3].

Several software for digital planning are being used to carry out the fabrication of the endodontic guide. Deviations are minimized by careful planning and printing. The accuracy of guide design can vary among different digital planning software. The merging technique of DICOM and STL files can also affect the accuracy of the guide [29]. The automatic merging of the two files by the software employed in this case may have contributed to the reduction in the accuracy of the guide. Artificial intelligence can be advantageous for segmenting CBCT scans and merging them with STL models [30]. The accuracy of the printed guide can be influenced by the thickness of the layers in the STL file. A thinner layer thickness results in a more reliable printed guide [28]. Using light-cured resin is typical in printing, and precise curing is essential to maintaining the dimensional stability of the guide. It is important to adjust printer settings based on manufacturer recommendations [31].

Ensuring the precise positioning of the guide on the teeth is highly important in clinical practice. To achieve this objective, verification windows were incorporated into the guide's design to visualize the correct placement in our study. In addition, rocking movements or an improper fit of the guide can jeopardize the accuracy of the treatment. Other solutions have also been suggested for better fitting of the guide. A custom-designed open-template guide using self-locking rod handles and lock arms that fit the target tooth and adjacent teeth has been proposed. Also, fixative pins can be used, especially in the posterior regions, due to the displacing forces of the tongue and muscles [32].

The use of metal sleeves over plastic sleeves is preferred because they provide better guidance for drilling and reduce the risk of damage to plastic due to overheating or unintended drilling [32]. The minimum height of the sleeve has been proposed as 5 mm in articles [24]. In this case report, a sleeve with a height of 4 mm was designed, which may be justifiable for error and deviation from the path in this study. It seems that the higher the height of the sleeve, the more contact area it has with the drill, and it can perform the guiding process with greater accuracy. Adaptation of the drill to the intended sleeve should also be considered for greater guiding accuracy. The Munce bur with a length of 16 mm and a diameter of 1 mm was used in the drilling process. The design of this bur includes a convergence at the beginning of a few millimeters from the tip of the bur, which due to the mismatch of this part with the sleeve, its accuracy is reduced, and it can be one of the other reasons for deviation from the path in this study. Therefore, a drill with a consistent diameter throughout all active areas in contact with the sleeve is recommended to reduce errors. Furthermore, it is important that the drill being used can withstand bending.

Guided endodontics has been reported as a safe and clinically practical method for less experienced operators [22, 33, 34]. In contrast, Fachin et al. reported that there is a significant difference between experienced and less experienced operators in fiberglass post removal in lower molars. This difference can be justified by an eccentric force exerted by the less experienced operator within the tolerance gap between the sleeve and drill, which may affect the accuracy [35].

It is important to keep in mind that in vitro studies, which employed guided endodontics for fiber post removal [7, 25, 35], used stable study models. However, the actual clinical practice condition differs somewhat. Clinicians should take this into account when interpreting the findings. The deviation observed in this study appears to be higher than that of previous studies. The reasons for deviation and solutions are summarized in Table 1.

## 4. Conclusion

This case report demonstrates the advantages of using static endodontic guides for removing the fiber post. Given the sensitivity of the fiber post removal procedure, precisely following the recommended guides for the construction of the guide as well as its clinical application is necessary to achieve a highly successful outcome. Any disruption in any of these stages can reduce the accuracy of the guide and cause iatrogenic errors.

## Figures and Tables

**Table 1:** Summary of possible sources of error in endodontic static guide construction and recommended solutions.

Sources of error	Recommendation
CBCT acquisition (DICOM format)	(1) Smallest possible field of view and ideally high resolution are required (2) Improve the quality of the CBCT image and enhance the diagnostic capacity by adjusting the acquisition settings, ensuring patient stability, and utilizing appropriate software for acquiring and running the images (3) Apply artifact reduction filters to preserve the details

Digital surface scan (STL format)	(1) Directly via an intraoral scanner (2) Digitizing a plaster cast using a laboratory scanner (3) No clinical alterations to the position and morphology of the teeth should be made after the CBCT and intraoral scanning process (4) New scans must be performed if the DICOM and STL files are of poor quality

Digital planning software	(1) Ensure that the software accurately synchronizes the DICOM file with the STL file using identifiable landmarks on the crown surfaces of the two files (2) Drill must be placed in the center of the root canal so that the tip reaches the end of the fiber post

3D printing	(1) Transform the STL file into a sequence of numerous layers or slices. Increase the reliability of the printed guide by reducing the thickness of the slices (2) Precise curing is essential to maintain the dimensional stability of the guide (3) Print endodontic static guides using light-curing liquid resin and direct light processing technology (4) Adjust printer settings based on manufacturer recommendations

Clinical application	(1) Ensure precise positioning of the guide on the teeth. Verification windows should be used in the guide's design to visualize the correct placement (2) Achieve greater occlusal surface adaptation to increase guide stability and reduce the risk of deviation (3) Rocking movements or improper fit of the guide can jeopardize the accuracy of the treatment (fixative pins can be used, especially in the posterior regions, due to the displacing forces of the tongue and muscles) (4) Use metal sleeves instead of plastic sleeves (5) Set the minimum height of the sleeve at 5 mm. (The higher the height of the sleeve, the more contact area it has with the drill, and it can perform the guiding process with greater accuracy) (6) Ensure that the metal sleeve is inserted properly into the guide template (7) Adaptation of the drill with the intended sleeve should be checked (8) A drill with a consistent diameter throughout all active areas in contact with the sleeve is recommended to reduce errors (9) The drill being used can withstand bending

## Data Availability

The data used to support the findings of this study are included within the article.
